# Supplementary material for: Imputing pre-diagnosis health behaviour in cancer registry data and investigating its relationship with oesophageal cancer survival time
Source: PLoS One. 2021 Dec 14;16(12):e0261416. doi: 10.1371/journal.pone.0261416 (PMC8670692; doi:10.1371/journal.pone.0261416)
Supplement: S1 Fig — (DOCX) [file pone.0261416.s001.docx]

S1 Fig. Flow chart of inclusions and exclusions of SEER oesophageal cancer cases

SEER oesophageal cancer cases from 2006 to 2015

*n*=39,233

Under 35 years of age

*n*=123

*n*=39,110

Missing survival time

*n*=512

*n*=38,598

Missing marital status (n=2,279) and/or race (n=121)

*n*=36,254

No BRFSS data records in same strata

*n*=606

*n*=35,648

Insufficient BRFSS records to provide two imputations

- Current smoker n=3,894
- Binge drinking n=3,974
- Heavy drinking n=3,977
- Physical activity n=3,890
- Obese n=3,930
- Current smoking with regular
  alcohol n=3,995

Included in the analysis

- Current smoking n=31,754
- Binge drinking n=31,674
- Heavy drinking n=31,671
- Physical activity n=31,758
- Obese n=31718
- Current smoking with regular alcohol n=31,653
