# Supplementary material for: Imputing pre-diagnosis health behaviour in cancer registry data and investigating its relationship with oesophageal cancer survival time
Source: PLoS One. 2021 Dec 14;16(12):e0261416. doi: 10.1371/journal.pone.0261416 (PMC8670692; doi:10.1371/journal.pone.0261416)
Supplement: S2 Fig — (DOCX) [file pone.0261416.s002.docx]

S2 Fig. Flow chart of inclusions and exclusions of BRFSS health behaviour data records

BRFSS data records from 2001 to 20010

*n*=3,469,905

Not from any of the 13 SEER States, *n*=2,515,009

*n*=954,896

Missing age (n=7,437), marital status (4,039) and/or race (n=9,463), total *n*=18,770

*n*=936,126

Cannot match^a^ age <30 *n*=96,729

*n*=839,397

Cannot match^a^ mixed race *n*=24,120

*n*=815,277

No SEER records in same strata, *n*=280,060

*n*=535,217

Missing behaviour data

- Current smoking n=3,477
- Binge drinking n=10,958
- Heavy drinking n=13,294
- Physical activity n=638
- Obese n=20,731
- Current smoking with regular alcohol n=16,017

Eligible donor records

- Current smoking n=531,740
- Binge drinking n=524,259
- Heavy drinking n=521,923
- Physical activity n=534,579
- Obese n=534,579
- Current smoking with regular alcohol n=519,200

Used in imputations

- Current smoker n=63,508 ● Physical activity n=63,516
- Binge drinking n=63,348 ● Obese n=63,436
- Heavy drinking n=63,342 ● Current smoker with regular alcohol n=63,306

^a^ All auxiliary variables could be coded identically in both SEER cancer registry data and BRFSS health behaviour data except the BRFSS data included an additional category for race. Unlike the SEER cancer registry data, the BRFSS data collection allowed respondents to describe their race as “mixed”. About 3.5% of BRFSS respondents selected this option. As these records did not match any SEER cancer registry records they did not contribute to the analysis. Similarly, with a minimum age of 35 years for SEER cancer cases and a 5-year lag, BRFSS respondents under 30 years of age could not match any SEER cancer registry records.
