# Supplementary material for: Imputing pre-diagnosis health behaviour in cancer registry data and investigating its relationship with oesophageal cancer survival time
Source: PLoS One. 2021 Dec 14;16(12):e0261416. doi: 10.1371/journal.pone.0261416 (PMC8670692; doi:10.1371/journal.pone.0261416)
Supplement: S3 Fig — (DOCX) [file pone.0261416.s003.docx]

S4 Fig. An example of Cox regression coefficient tending towards negative infinity.

As more than 5 of the 100 data sets have returned extreme estimates of the coefficients, these results are rejected. This specific example shows the estimated Cox regression coefficients for the behaviour ‘Current tobacco smoking with regular alcohol’ after age-standardisation.
