# Supplementary material for: Imputing pre-diagnosis health behaviour in cancer registry data and investigating its relationship with oesophageal cancer survival time
Source: PLoS One. 2021 Dec 14;16(12):e0261416. doi: 10.1371/journal.pone.0261416 (PMC8670692; doi:10.1371/journal.pone.0261416)
Supplement: S5 Fig — (DOCX) [file pone.0261416.s005.docx]

S6 Fig. Age-standardised hazard ratios for simulated smoking in adenocarcinoma subgroup.

The bar chart shows the number of data sets where the algorithm has returned extreme values (<0.01 or >100) for estimated hazard ratios. The second chart shows the median estimated hazard ratio and associated 95% empirical confidence intervals for those behaviours which have recorded 5 or less extreme hazard ratios out of 100.
