# Supplementary material for: Imputing pre-diagnosis health behaviour in cancer registry data and investigating its relationship with oesophageal cancer survival time
Source: PLoS One. 2021 Dec 14;16(12):e0261416. doi: 10.1371/journal.pone.0261416 (PMC8670692; doi:10.1371/journal.pone.0261416)
Supplement: S6 Fig — (DOCX) [file pone.0261416.s006.docx]

S7 Fig. The proportion of data sets failing to converge (HR<0.01 or HR>100) when using I2C2 with age-stratified simulated smoking.
