# Supplementary material for: Imputing pre-diagnosis health behaviour in cancer registry data and investigating its relationship with oesophageal cancer survival time
Source: PLoS One. 2021 Dec 14;16(12):e0261416. doi: 10.1371/journal.pone.0261416 (PMC8670692; doi:10.1371/journal.pone.0261416)
Supplement: S7 Fig — (DOCX) [file pone.0261416.s007.docx]

S8 Fig. Age-stratified hazard ratios for simulated smoking in oesophageal cancer.

The first CI in each pair shows the HRs for ‘true’ smoking status and the second shows the results obtained using the imputed smoking status with misclassification correction. Where more than 5 in 100 data sets returned a HR<0.01 or HR>100, results are suppressed. The I2C2 algorithm fails to fully correct for the attenuation of results towards the null in any of the age categories.

Target HR=2.00
