# Supplementary material for: Imputing pre-diagnosis health behaviour in cancer registry data and investigating its relationship with oesophageal cancer survival time
Source: PLoS One. 2021 Dec 14;16(12):e0261416. doi: 10.1371/journal.pone.0261416 (PMC8670692; doi:10.1371/journal.pone.0261416)
Supplement: S8 Fig — (DOCX) [file pone.0261416.s008.docx]

S9 Fig. Simulation of the relationship between sample size, agreement beyond chance rate and estimated hazard ratios for smoking status.

Target HR=2.00

The black solid line connects median estimated hazard ratios for ‘true’ smoking status with the black dotted lines representing the associated 95% confidence intervals. The red solid line connects median hazard ratios estimated from ‘imputed’ smoking status using the correct score estimation misclassification correction. The red dotted lines connect the associated 95% confidence intervals.

S9 Fig (cont). Simulation of the relationship between sample size, agreement beyond chance rate and estimated hazard ratios for smoking status.

Target HR=1.50

The black solid line connects median estimated hazard ratios for ‘true’ smoking status with the black dotted lines representing the associated 95% confidence intervals. The red solid line connects median hazard ratios estimated from ‘imputed’ smoking status using the correct score estimation misclassification correction. The red dotted lines connect the associated 95% confidence intervals.

S9 Fig (cont). Simulation of the relationship between sample size, agreement beyond chance rate and estimated hazard ratios for smoking status.

Target HR=1.25

The black solid line connects median estimated hazard ratios for ‘true’ smoking status with the black dotted lines representing the associated 95% confidence intervals. The red solid line connects median hazard ratios estimated from ‘imputed’ smoking status using the correct score estimation misclassification correction. The red dotted lines connect the associated 95% confidence intervals.

S9 Fig (cont). Simulation of the relationship between sample size, agreement beyond chance rate and estimated hazard ratios for smoking status.

Target HR=1.00

The black solid line connects median estimated hazard ratios for ‘true’ smoking status with the black dotted lines representing the associated 95% confidence intervals. The red solid line connects median hazard ratios estimated from ‘imputed’ smoking status using the correct score estimation misclassification correction. The red dotted lines connect the associated 95% confidence intervals.

S9 Fig (cont). Simulation of the relationship between sample size, agreement beyond chance rate and estimated hazard ratios for smoking status.

Target HR=0.80

The black solid line connects median estimated hazard ratios for ‘true’ smoking status with the black dotted lines representing the associated 95% confidence intervals. The red solid line connects median hazard ratios estimated from ‘imputed’ smoking status using the correct score estimation misclassification correction. The red dotted lines connect the associated 95% confidence intervals.

S9 Fig (cont). Simulation of the relationship between sample size, agreement beyond chance rate and estimated hazard ratios for smoking status.

Target HR=0.67

The black solid line connects median estimated hazard ratios for ‘true’ smoking status with the black dotted lines representing the associated 95% confidence intervals. The red solid line connects median hazard ratios estimated from ‘imputed’ smoking status using the correct score estimation misclassification correction. The red dotted lines connect the associated 95% confidence intervals.

S9 Fig (cont). Simulation of the relationship between sample size, agreement beyond chance rate and estimated hazard ratios for smoking status.

Target HR=0.50

The black solid line connects median estimated hazard ratios for ‘true’ smoking status with the black dotted lines representing the associated 95% confidence intervals. The red solid line connects median hazard ratios estimated from ‘imputed’ smoking status using the correct score estimation misclassification correction. The red dotted lines connect the associated 95% confidence intervals.
