# Supplementary material for: Imputing pre-diagnosis health behaviour in cancer registry data and investigating its relationship with oesophageal cancer survival time
Source: PLoS One. 2021 Dec 14;16(12):e0261416. doi: 10.1371/journal.pone.0261416 (PMC8670692; doi:10.1371/journal.pone.0261416)
Supplement: S1 Table — (DOCX) [file pone.0261416.s009.docx]

S3 Table. Number of SEER oesophageal cancer cases seeking donor records for current smoking behaviour and the proportion of these failing to obtain two donor records.

|  | Seeking Donor Records | | Failed to Obtain 2 Donor Records | |
| --- | --- | --- | --- | --- |
|  | Frequency | % of total | Frequency | % of group |
| Total | 36,254 | 100.0% | 4,500 | 12.4% |
| Cancer type  ESCC  EAC | 11,694  20,354 | 32.3%  56.1% | 1,616  2,282 | 13.8%  11.2% |
| Year  2006  2007  2008  2009  2010  2011  2012  2013  2014  2015 | 3,462  3,525  3,553  3,693  3,579  3,608  3,653  3,649  3,716  3,816 | 9.5%  9.7%  9.8%  10.2%  9.9%  10.0%  10.1%  10.1%  10.2%  10.5% | 693  661  622  731  508  426  501  150  100  108 | 20.0%  18.8%  17.5%  19.8%  14.2%  11.8%  13.7%  4.1%  2.7%  2.8% |
| Age group  35-39 years  40-44 years  45-49 years  50-54 years  55-59 years  60-64 years  65-69 years  70-74 years  75-79 years  80+ years | 206  535  1407  2684  4337  5553  5726  4888  4467  6451 | 0.6%  1.5%  3.9%  7.4%  12.0%  15.3%  15.8%  13.5%  12.3%  17.8% | 2  7  28  148  385  725  828  751  745  881 | 1.0%  1.3%  2.0%  5.5%  8.9%  13.1%  14.5%  15.4%  16.7%  13.7% |
| Sex  Male  Female | 28274  7980 | 78.0%  22.0% | 4190  310 | 14.8%  3.9% |
| Race  White  Black  Asian or Pacific Islander  American Indian or Alaska Native | 30619  3783  1640  212 | 84.5%  10.4%  4.5%  0.6% | 3164  727  515  94 | 10.3%  19.2%  31.4%  44.3% |
| Marital status  Married (incl common law)  Divorced  Widowed  Single (Never married) | 20621  4584  4860  6189 | 56.9%  12.6%  13.4%  17.1% | 2275  301  532  1392 | 11.0%  6.6%  10.9%  22.5% |
| State of residence  Alaska^a^  California^b^  Connecticut  Georgia  Hawaii  Iowa  Kentucky  Louisiana  Michigan  New Jersey  New Mexico  Utah  Washington | 48  13209  2024  4046  533  1946  2384  2209  2129  4087  738  676  2225 | 0.1%  36.4%  5.6%  11.2%  1.5%  5.4%  6.6%  6.1%  5.9%  11.3%  2.0%  1.9%  6.1% | 48  3625  38  355  55  38  52  86  70  87  11  10  27 | 100.0%  27.4%  1.9%  8.8%  10.3%  2.0%  2.2%  3.9%  3.3%  2.1%  1.5%  1.5%  1.2% |
| Follow-up status  Censored  Died | 8705  27549 | 24%  76% | 608  3892 | 7.0%  14.1% |

^a^ This is a small cancer registry specific to Alaska Natives.

^b^ Given the large population of California the number oesophageal cancer cases was higher, but the number of BRFSS health surveys is constant for each State.
